# Ion spectroscopy in methane activation

**DOI:** 10.1002/mas.21698

**Published:** 2021-05-19

**Authors:** Jana Roithová, Joost M. Bakker

**Affiliations:** ^1^ Department of Spectroscopy and Catalysis Radboud University Nijmegen Nijmegen The Netherlands; ^2^ Radboud University, Institute for Molecules and Materials FELIX Laboratory Nijmegen The Netherlands

**Keywords:** argon tagging, ion spectroscopy, metal cations, methane activation, reaction intermediates

## Abstract

This review is devoted to ion spectroscopy studies of complexes relevant for the understanding of methane activation with metal ions and clusters. Methane activation starts with the formation of a complex with a metal ion. The degree of the interaction between an intact methane molecule and the ion can be monitored by the perturbations of C–H stretch vibrations in the methane molecule. Binding mediated by the electrostatic interaction results in a η^3^ type coordination of methane. In contrast, binding governed by orbital interactions results in a η^2^ type coordination of methane. We further review the spectroscopic characterization of activation products of metal–methane reactions, such as the metal–carbene and carbyne products resulting from the interaction of selected 5d metals with methane. The focus of recent research in the field has shifted towards the investigation of interactions between methane and metal clusters. We show examples highlighting that metal clusters can be more reactive in methane activation reactions.

## 1 INTRODUCTION

The field of gas phase ion chemistry has greatly contributed to our knowledge about reactions in the atmosphere (Farnik & Lengyel, 2018; Klemperer & Vaida, 2006; Laskin et al., 2018), in interstellar space (Bohme, 2011, 2016; Wakelam et al., 2010; Zhao et al., 2020), and alike media (Laskin et al., 2013). Apart from describing processes naturally occurring in the gas phase, gas‐phase ion chemistry also serves as a tool for describing principles governing processes in the condensed phase or at the interface.

The advantage of gas‐phase ion chemistry is that it offers methods to investigate molecules and ions under well‐defined conditions. Usually, the internal and kinetic energy of the ionic, as well as neutral reactants, are known and the reactions are controlled to proceed in bimolecular collisions. These conditions offer an ideal case for high‐level theoretical studies. In the end, the synergy between the defined gas‐phase ion chemistry experiments and the theoretical calculations offers a detailed insight into the fundamentals of chemical reactivity (Schwarz, 2017a, 2017b).

An example of a chemistry field that profited from model studies in the gas phase is the field of metal catalysis. It was the model studies in the gas phase that discovered two‐state and multi‐state reactivity of transition metals. The gas‐phase studies also enlightened the principles of methane activation (and C–H activation in general) by metals, metal oxides, metal complexes, metal clusters, and others (Armentrout, 2017; Bohme & Schwarz, 2005; Dietl et al., 2012; Roithova & Schröder, 2010; Schwarz, 2011, 2014; Schwarz et al., 2017; Zhou et al., 2016). Much like for methane activation, gas‐phase studies described principles of activation, reactivity, and transformation of other small molecules too (Blagojevic et al., 2019; Muramatsu & Tsukuda 2019). As a result, we obtained a detailed knowledge of molecular transformations and on whole catalytic cycles (Bozovic et al., 2010).

**Scheme 1:**
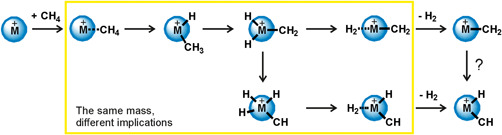
Methane dehydrogenation upon reaction with a metal cation

This review will focus on the spectroscopic investigation of structures of ion–molecule complexes and intermediates in ion–molecule reactions. To demonstrate why spectroscopy is a key tool in investigating details of molecular interactions, we will show spectroscopic details of methane activation at a metal cation center (Scheme 1). The metal cation and methane initially form an ion–molecule complex. Within this complex, the hydrogen atoms can sequentially migrate from the carbon atom to the metal. The reaction sequence can finish by elimination of H_2_ if this dissociation limit is below the initial energy of the reactants. The remaining ionic product can either be a metal–carbene (M^+^CH_2_), a metal carbyne hydride (HM^+^CH), or even a metal carbide dihydride (H_2_M^+^C) cation (not shown in Scheme 1).

**Figure 1:**
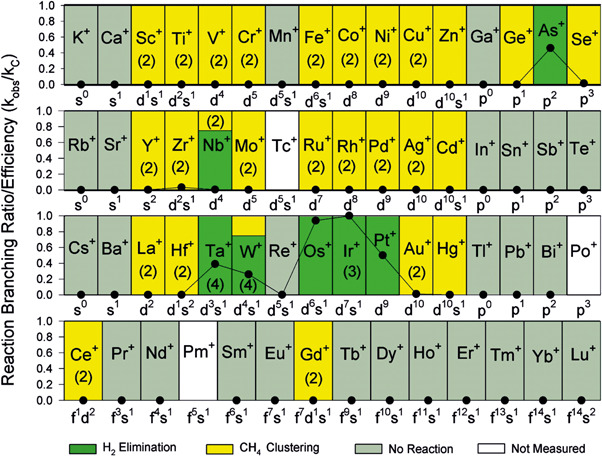
Periodic variations observed in the reaction efficiency (*k*/*k*_c_) (*k*_c_ is the collisional rate constant), represented by solid circles, for the reactions of ground‐state atomic cations with CH_4_. The numbers in parentheses indicate the number of sequential color‐coded reactions observed. Reactions of Tc^+^, Pm^+^, and Po^+^ were not investigated. Reprinted with permission from reference Shayesteh et al. (2009). Copyright (2009) American Chemical Society [Color figure can be viewed at wileyonlinelibrary.com]

Diethard Bohme and his colleagues in 2009 made an extensive survey of the reactivity of metal cations toward methane (Figure 1) (Shayesteh et al., 2009). They have shown that the full reaction sequence in Scheme 1 can be accomplished with cations of only seven elements in the periodic table (green in Figure 1). The mechanistic and electronic aspects of these results were discussed many times, therefore we will not explain them here. We will rather focus on the spectroscopy of these species.

The metal cations shown in yellow did not show any dehydrogenation reaction in thermal collisions with methane. Their reactions ended by the formation of metal cation–methane adducts. However, classical mass spectrometry cannot differentiate between the possible structures of these adducts (the yellow frame in Scheme 1 indicates isobaric structures) and therefore cannot obtain further details about the reactivity of these metal cations with methane. Ion spectroscopy can obtain spectroscopic (infrared [IR], ultraviolet [UV], visible [vis]) signatures of the ions and thus can make a step further. In the following, we will explain shortly the tools of ion spectroscopy and their application in investigating of mechanisms of small molecules activation.

## 2 METHODS OF ION SPECTROSCOPY

For the structure determination of products resulting from reactions of metal ions and clusters with methane, ion spectroscopy has established itself as the method of choice, because the typical densities of the methane activation products are usually too low to enable direct absorption techniques. In addition, ion spectroscopy offers information based on the photofragmentation chemistry. The combination of laser sources with mass‐spectrometric detection (and purification) techniques has proven itself as a mature technique for structure determination of virtually any polyatomic ion that can be brought into or formed in the gas phase (Asmis, 2012; Baer & Dunbar, 2010; Bieske & Dopfer, 2000; Duncan, 2000; Maitre et al., 2020; Rijs & Oomens, 2014; Roithova, 2016; Schwarz & Asmis, 2019; Stedwell et al., 2013). The most direct spectroscopic technique in terms of molecular structure analysis is IR spectroscopy, which probes the vibrations, making it sensitive to both local and global molecular structures. In the most straightforward implementation, an ion is irradiated with laser light in resonance with an IR‐active vibration, and the absorption of one or more photons results in the fragmentation of the ion. Mass‐spectrometric observation of the fragmentation then signals the presence of a resonant photon absorption of the ions under study. If the energy required for the fragmentation within the time‐scale of the experiment is lower than that carried by a single IR photon, a resulting IR photodissociation (IR‐PD) spectrum is very similar to the linear absorption spectrum. However, if the energy required exceeds that of the IR photon, a sequential absorption of multiple IR photons by the IR active coordinate can take place if the absorbed energy is efficiently dissipated in the bath of vibrational coordinates of the ion through intramolecular vibrational redistribution (Jašíková & Roithova, 2018; MacAleese & Maitre, 2007; Oomens et al., 2006). Although the relationship between a so‐called IR multiple photon dissociation (IR‐MPD) spectrum and the linear absorption spectrum is more complex than for an IR‐PD spectrum, the resulting spectral fingerprint is sufficiently close that the structure determination is often unambiguous. Midway both lies the approach to form so‐called messenger complexes, where the ion of interest is tagged with a weakly bound species, such as He or Ar atoms (Asmis, 2012; Bieske & Dopfer, 2000; Gerlich, 2018; Schwarz & Asmis, 2019; Wolk et al., 2014). Here, the influence of the attached atom on the ion's structure should be as low as possible (Roithova et al., 2016), and with the low binding energy multiple‐photon effects on the spectra are reduced.

IR spectra obtained by IRPD as well as IR‐MPD techniques can qualitatively be interpreted using known frequencies for vibrations of functional groups. However, the identification typically relies on a comparison with simulated IR spectra, calculated for trial structures using density functional theory calculations. With a plethora of exchange‐correlation functionals to choose from, the most popular functionals used remain PBE and B3LYP (Becke, 1988; Lee et al., 1988; Perdew et al., 1996; Vosko et al., 1980).

Some of the spectroscopic characterizations were done with the UV/vis photodissociation technique (Antoine & Dugourd, 2011; Förstel et al., 2019; Srnec et al., 2018). The experimental setup is analogous as described for vibrational photodissociation spectroscopy, but the photon wavelength allows probing electronic transitions. Electronic excitation is usually followed by internal conversion to the highly vibrationally excited ground state followed by dissociation. For some, often small ions, photodissociation at the excited state occurs faster than the internal conversion (van der Linde et al., 2013). Similar to IR spectra, UV/vis spectra of isolated ions are usually assigned based on theory. The methods are often based on time‐dependent density functional theory (DFT) calculations. We do not discuss results obtained by this method in detail, but we do list relevant studies in the tables.

The reactions of metal ions or clusters with methane or other substrates, such as cyclopropane C_3_H_6_ or ethylene oxide C_2_H_4_O, that lead to relevant activation products, can be done in different environments. In the simplest experiments, the ions of interest are formed via pulsed laser ablation in the presence of either pure methane or methane diluted in an inert gas injected via a pulsed valve. [M,C,4H]^+^ ions are then formed in the expansion and vibrationally cooled. Ion or cluster formation and reaction can also be separated by executing ablation in neat helium, and confining the formed ions in a flow tube‐type reaction cell, where methane is introduced via a second pulsed valve, either pure or diluted. In the latter scenario, at least some form of thermalization has taken place ensuring the ablation process does not influence the reaction. For the preparation of the individual complex, we refer the readers to the original literature.

## 3 INTERMEDIATES IN METHANE ACTIVATION

Selective transformation of methane into methanol would enable a more efficient utilization of methane stocks. However, finding the way for the selective transformation that would avoid competing processes such as overoxidation still represents a formidable research challenge. A popular line of this study involves methane oxidation on metals. The oxidation starts with the activation of the C–H bond. A huge effort was devoted into understanding which metals could mediate this reaction step and what the mechanistic background is (Armentrout, 2017; Dietl et al., 2012; Roithova & Schröder, 2010; Schwarz, 2011, 2014; Schwarz et al., 2017). Here, we will review the contribution of ion spectroscopy to the build‐up of knowledge of methane activation by metal ions and metal clusters.

The word “activation” can have a broad meaning. We use the word in a sense of the activation leading to the breaking of a C–H bond. It could also be used for the mere weakening of a C–H bond by the interaction between a metal and methane; however, we choose to denote this interaction as complexation or solvation of a metal by methane molecules. We include the formation of the methane–metal complexes to this review as well because their understanding is important for understanding the overall C–H activation pathway.

### 3.1 [Metal(CH_4_)_*n*_]^+/−^ complexes

Metal cations initially bind with methane via σ‐bonding with the C–H bonds. Electron donation of the C–H bond to the metal (and potentially back‐donation from the metal to the σ* orbital of the C–H bond) results in a weakening of the C–H bond. The weakening of the bond is associated with a red shift of the corresponding C–H stretching vibration. Consequently, it has become a standard to compare the detected C–H vibrations in metal–methane complexes with the vibrational modes of the free methane molecule. Methane has four fundamental vibrational modes, the symmetric (*ν*_1_, 2917 cm^−1^) and anti‐symmetric (*ν*_3_, 3019 cm^−1^) C–H stretching vibrations, and two deformation modes (*ν*_2_ and *ν*_4_) in the region around 1500 cm^−1^.

Formation of an adduct between a metal ion and methane is the first step towards methane activation (see Table 1 for the investigated metal–ion complexes with methane). This association step can be followed by an insertion of the metal ion into the C–H bond. The insertion step requires that the metal ion is redox active. Hence, no insertion into the C–H bond is expected for main‐group metal ions in their preferred oxidation state. Accordingly, in investigating methane complexes of Li^+^, Rodrigues and Lisy (2011b) only detected metal ions “solvated” by methane molecules. The study shows how the charged center affects molecules in a sequential filling of the first and the second solvation shell. The structure of the first solvation shell has been investigated by argon tagging photodissociation spectroscopy. The second solvation shell could be studied by direct photodissociation because the IR photons have sufficient energy to induce the elimination of the molecules from the second coordination shell.

**Table 1:** Ion spectroscopy of metal ion–methane complexes

Complexes	*m*	Type of ion spectroscopy	Spectral range (cm^−1^)^a^	ν_1_ of the first coordinated CH_4_ b (cm^−1^)	References
Li(CH_4_)_*m*_^+^	1–9	Argon tagging, IRPD	2800–3100	2859 ([Li(CH_4_)_1_Ar]^+^)	Rodriguez and Lisy (2011a)
Li(CH_4_)^+^	1–6	Argon tagging	2800–3100	2859 ([Li(CH_4_)_1_Ar]^+^)	Rodriguez and Lisy (2011b)
Mg(CH_4_)^+^	1	UVPD	310–342 nm		Cheng et al. (1996)
Ca(CH_4_)^+^	1	UVPD	22,100– 23,000		Chen et al. (1997)
Al(CH_4_)_*m*_^+^	1–6	IRPD	2800–3100	2850	Poad et al. (2008)
V(CH_4_)^+^	1	UVPD	15,800– 16,600		Hayes et al. (1997)
Mn(CH_4_)_*m*_^+^	1–6	IRPD	2700–3100	2836	Dryza and Bieske (2010)
Fe(CH_4_)_*m*_^+^	1–4	IR(M)PD	2500–3200	2646	Citir et al. (2010)
Co(CH_4_)_*m*_^+^	1–4	Argon tagging	2500–3100	2547 ([Co(CH_4_)Ar_2_]^+^)	Kocak et al. (2014)
Ni(CH_4_)_*m*_^+^	1–4	Argon tagging	2500–3100	2597 ([Ni(CH_4_)Ar_2_]^+^)	Kocak et al. (2014)
Cu(CH_4_)_*m*_^+^	1–6	Argon tagging, IRMPD	2500–3100	2635 ([Cu(CH_4_)Ar_2_]^+^)	Kocak et al. (2015)
Zn(CH_4_)^+^	1	UVPD	38,000–48,000		Lu et al. (2001)
Zr(CH_4_)_*m*_^+^	1–4	Argon tagging, IRMPD	2550–3100	2730	Kozubal et al. (2020)
Ag(CH_4_)_*m*_^+^	1–6	IRMPD	2500–3100	2738	Kocak et al. (2015)
Au(CH_4_)_*m*_^+^	3–8	Argon tagging	2800–3200		Gentleman et al. (2018)
[PtCH_4_](−)	1 ‐‐> H–Pt–CH_3_–	Photoelectron			Liu et al. (2019)

Abbreviations: IRMPD, infrared multiple photon dissociation; UVPD, ultraviolet photodissociation.

^a^ If not stated otherwise.

^b^ ν_1_ (free CH_4_) = 2917 cm^−1^; *ν*_3_ (free CH_4_) = 3019 cm^−1^.

**Figure 2:**
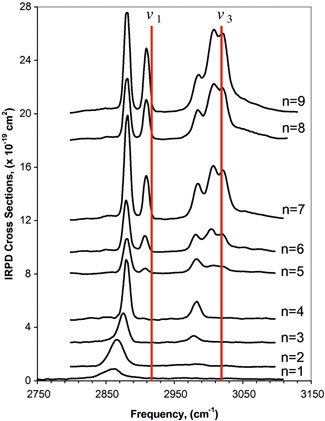
Infrared photodissociation spectra of [Li(CH_4_)_*n*_]^+^ (*n* = 1–9). For *n* = 1–4, the studied ions were tagged by argon. New features beginning at *n* = 5 indicate the onset of a second solvent shell. Reprinted from reference Rodriguez and Lisy (2011a), Copyright (2010), with permission from Elsevier [Color figure can be viewed at wileyonlinelibrary.com]

The first solvation shell of the lithium cation is formed by four methane molecules. The IR spectra of [Li(CH_4_)_*n*_]^+^ (*n* = 1–4) (the bottom four spectra in Figure 2) show two bands in the range of the C–H stretching vibrations. These bands correspond to the symmetric and antisymmetric stretching of methane. Note that the polarization of methane molecules by the lithium ion (or any ion discussed below) permits detection of the (otherwise dipole‐forbidden) symmetric stretch vibration. The detected signatures agree with a theoretical prediction of the interaction of the methane molecule with the lithium ion in the η^3^ fashion. The detected bands are red‐shifted with respect to the frequencies of the isolated methane molecule (the red lines in Figure 2). The red shift of the symmetric vibration decreases with the filling of the first solvation shell (up to *n* = 4), and then stabilizes.

The second solvation shell presents itself by new bands in the IR spectra (top five spectra in Figure 2). The symmetric stretch of the methane molecules in the second solvation shell is only slightly red‐shifted compared to *ν*_1_ of the free methane molecule and the number of molecules in the second solvation shell does not affect the frequency of this vibration. The antisymmetric stretch is represented by two bands almost centered around the original triply degenerate *ν*_3_ band of the free methane molecule. Again, the bands are not affected by the number of molecules in the second coordination shell. This tells us that the central ion's interaction with methane molecules is limited beyond the first coordination shell.

Another example of a main‐group metal ion is Al^+^ (Poad et al., 2008). The preferred oxidation state of aluminum is +III, hence we could expect that the singly charged aluminum ion might be prone to the oxidative addition to the C–H bond of methane. In fact, theory predicts that this process should be favorable, but there is no spectroscopic evidence. Instead, the IR spectra of the [Al(CH_4_)_*n*_]^+^ complexes (*n* = 1–6) show a very similar signature as the methane molecules in the first coordination shell of Li^+^ (Figure 2). This suggests that they are intact and η^3^‐coordinated. The aluminum ion is larger than Li^+^, and, accordingly, the authors found that six methane molecules fit into its first coordination shell (Poad et al., 2008). Analogous characteristics were also found for complexes with manganese [Mn(CH_4_)_*n*_]^+^ (*n* = 1–6) (Dryza & Bieske, 2010).

**Figure 3:**
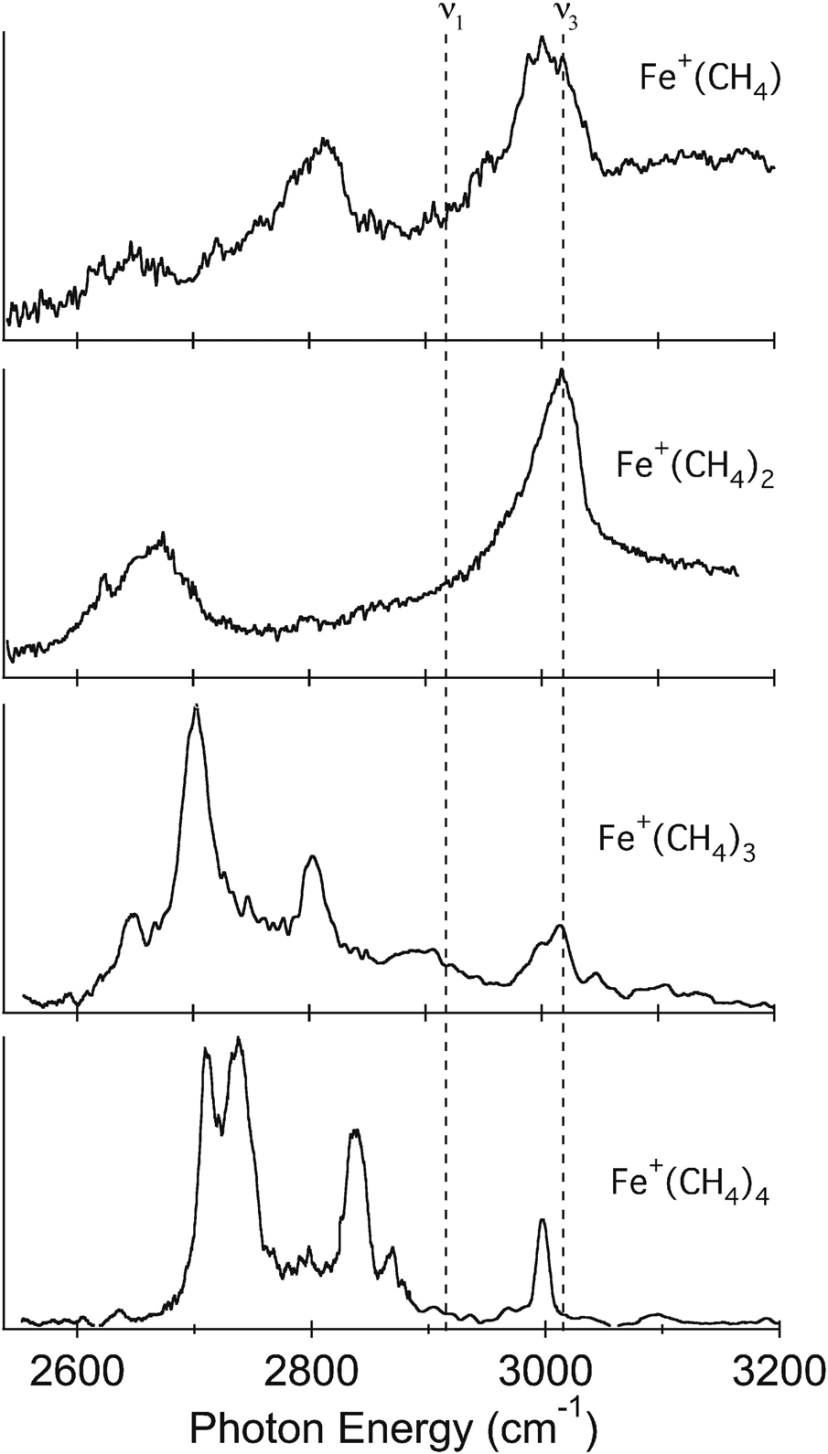
Infrared photodissociation spectra of Fe^+^(CH_4_)_*n*_ (*n* = 1−4) in the C−H stretching region. The vertical lines indicate the positions of the symmetric (*ν*_1_ = 2917 cm^−1^) and antisymmetric stretch (*ν*_3_ = 3019 cm^−1^) in CH_4_. Reprinted with permission from reference Citir et al. (2010). Copyright (2010) American Chemical Society

The examples of metal–methane complexes listed above reveal a regular build‐up of the solvation shell. Each next methane molecule is bound with a somewhat smaller binding energy and the IR spectroscopic signatures gradually change. The iron cation is the first cation in the first row that shows irregularities in the formation of the first solvation shell (Citir et al., 2010). Here, the binding energies of the coordinated methane molecules do not gradually decrease with their number as in the previous cases. The second methane molecule, for instance, has a larger binding energy than the first one (Schultz & Armentrout, 1993; Zhang et al., 2001), even though both are η^3^ coordinated. This is reflected in a larger redshift of the symmetric stretching vibration for [Fe(CH_4_)_2_]^+^ than for [Fe(CH_4_)]^+^ (Figure 3). Adding the third and fourth methane molecules leads to a significant change in the appearance of the IR spectra. The bands for [Fe(CH_4_)_3_]^+^ and [Fe(CH_4_)_4_]^+^ are consistent with a change of the coordination of all methane molecules to η^2^. In [Fe(CH_4_)_3_]^+^, the three methane molecules coordinate trigonally, whereas in [Fe(CH_4_)_4_]^+^ the four methane molecules can coordinate either in a tetrahedral or a square planar geometry with only minimal energy differences.

The iron–methane complexes were not investigated using the argon tagging method, because the (calculated) binding of argon to the iron cation was deemed to influence the vibrational structure of the Fe^+^(CH_4_)_*n*_ (*n* = 1−4) spectra too much. Instead, the authors used IR‐MPD spectroscopy for untagged complexes (which led to a lower resolution of the IR spectra; see Figure 3). This strategy was re‐evaluated in the subsequent studies of methane complexes with Co^+^ and Ni^+^. Because these cations bind the “solvating” molecules even stronger, the binding energy of the first and the second methane molecules is too large to permit the simple IRMPD approach with the available laser sources. Therefore, the authors relied on tagging using two argon atoms for Co^+^(CH_4_) and Ni^+^(CH_4_) (Kocak et al., 2014).

The IR spectra of [Co(CH_4_)Ar_2_]^+^ and [Ni(CH_4_)Ar_2_]^+^ are consistent with η^2^ coordination of the methane molecule. The theoretical analysis of these complexes suggests that this binding motif is due to the properties of the metal cations and not due to the coordination of additional argon atoms. In fact, the iron cation is a borderline case that prefers η^3^ coordination if only 1 or 2 methane molecules are coordinated. Beyond iron, all late‐transition metal cations coordinate methane molecules as η^2^‐ or even η^1^‐ligands. This coordination is associated with higher binding energies and has a more covalent character (Citir et al., 2010; Kocak et al., 2014), which is reflected in the increasingly red‐shifted frequency for the lowest‐lying C–H stretch vibration (see Table 1). However, there is no direct correlation between the red shift of this IR band and the binding energy of the corresponding methane molecules: the stretching vibration reflects the perturbation of the given C–H bond(s) which not only depends on binding strength but also strongly on the electronic configuration of the central metal (Citir et al., 2010; Kocak et al., 2014).

**Figure 4:**
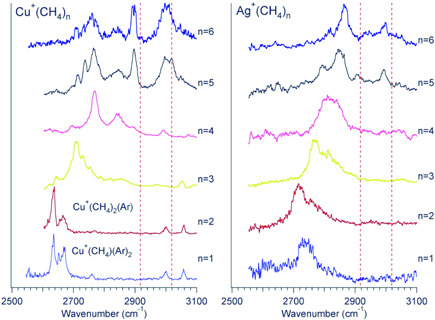
Vibrational spectra of Cu^+^(CH_4_)(Ar)_2_, Cu^+^(CH_4_)_2_(Ar), and Cu^+^(CH_4_)_3−6_, (left) and Ag^+^(CH_4_)_*n*_ (*n* = 1−6) (right). The *y*‐axis shows the normalized photofragment yield. The dashed vertical lines show the positions of the bare CH_4_ stretches (2917 and 3019 cm^−1^). Reprinted with permission from reference Kocak et al. (2015). Copyright (2015) American Chemical Society [Color figure can be viewed at wileyonlinelibrary.com]

Spectra of methane complexes with a copper cation nicely show the weak correlation between the C–H stretching frequency and the binding energy between the metal and a methane molecule (Figure 4) (Kocak et al., 2015). The interaction energy between Cu^+^ and the methane molecules is similar to those in Co^+^ and Ni^+^ complexes and for all these complexes, the methane molecules are η^2^ coordinated in the first solvation shell. Yet, the red shift of the symmetric C–H vibration is significantly smaller for the copper complexes than for the cobalt and nickel complexes.

This is rationalized by a symmetric filling of the Cu^+^ coordination sphere (Kocak et al., 2015). The first two methane molecules coordinate linearly. The coordination then changes to trigonal with the third ligand. With the fourth methane molecule, the coordination sphere becomes less rigid, because the tetrahedral, trigonal pyramidal, and square planar coordination geometries have only minimal energy differences. Although the spectroscopic differences between these geometrical variants of the [Cu(CH_4_)_4_]^+^ complex are also small, the tetrahedral coordination seems to dominate the experimental spectrum. The fifth methane molecule starts to fill the second coordination shell. This is clearly demonstrated in the spectrum by intense bands at about 2900 and 3000 cm^−1^, frequencies only slightly red‐shifted from the free methane vibrations (see Figure 4, left panel, *n* = 5 and 6). The spectral signature of the second solvation shell is analogous to that described above for the lithium complexes (Figure 2).

The methane complexes were investigated for the whole coinage metal group, offering an interesting comparison. The silver cation is larger than the copper cation and, consequently, its binding distances to the methane molecules are larger (Kocak et al., 2015). This is associated with a smaller binding energy, with a smaller perturbation of the C–H bonds, and thus with a smaller red‐shift of the symmetric C–H stretch bands (Figure 4). The second consequence of the larger diameter of silver is that the first coordination sphere can be filled with up to six methane molecules. The filling of the first coordination shell is reflected by a gradual change of the band positions in the spectra. Unlike for the copper complexes, the spectra do not show any abrupt appearance of the signature of the second solvation shell. However, weak additional bands at about 3000 cm^−1^ appear in the spectra of [Ag^+^(CH_4_)_*n*_] with *n* = 5 and 6. These bands suggest that these complexes can also have isomers with four methane molecules in the first coordination shell and the fifth and sixth in the second coordination shell.

**Figure 5:**
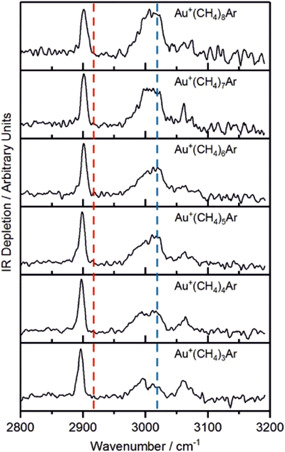
Infrared (IR) multiple photon dissociation spectra of Au^+^(CH_4_)_*n*_Ar (*n* = 3–8). The dashed lines indicate the positions of the bare CH_4_ stretches. Copyright authors (CC BY license) (Gentleman et al., 2018) [Color figure can be viewed at wileyonlinelibrary.com]

For the gold cation, spectroscopic data are only available for complexes with three and more methane molecules (Figure 5) (Gentleman et al., 2018). For all complexes investigated ([Au^+^(CH_4_)_*n*_], *n* = 3–8), only bands that correspond to the second solvation shell were detected. This is in accordance with the strong preference of gold(I) complexes for linear coordination in the first solvation shell. Hence, already the third methane molecule starts to fill the second coordination shell. An interesting question is then why the spectra do not show any signature of vibrations of the methane molecules in the first coordination shell. The authors made an extensive theoretical investigation and showed that the methane molecules can freely rotate around the Au–C bond axis, which then leads to a broadening of the C–H stretching vibrational band up to the point that no resolved band is seen.

**Figure 6:**
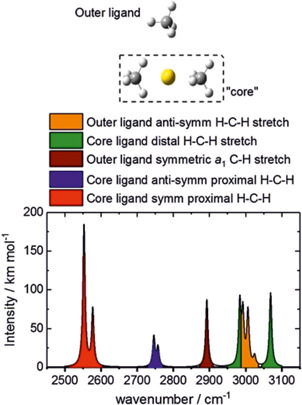
Structure of the Au^+^(CH_4_)_3_ complex with the staggered core structure and its assigned simulated infrared spectrum. Copyright authors (CC BY license) (Gentleman et al., 2018) [Color figure can be viewed at wileyonlinelibrary.com]

Figure 6 shows one of the localized geometries of [Au^+^(CH_4_)_3_] with two methane molecules in a staggered geometry in the first coordination shell. The corresponding IR spectrum shows that the third, outer methane molecule is responsible for the bands at about 2900 and 3000 cm^−1^. All other bands originate from the CH_4_ molecules in the first coordination shell. The change of the staggered geometry to the eclipsed configuration of the two methane molecules leads to a change of the stretching frequencies of the C–H bonds interacting with gold (the “proximal” C–H bonds), resulting in the red and blue bands in Figure 6. The two conformers are almost isoenergetic and are separated by only a minimal energy barrier. Hence, the authors speculated on a free mutual rotation of the methane molecules in the first coordination shell, which could lead to a broadening of the vibrational bands associated with the proximal C–H bonds. This extensive broadening was deemed the reason for not detecting these bands (i.e., the red and blue bands in Figure 6) in the experimental spectrum.

**Figure 7:**
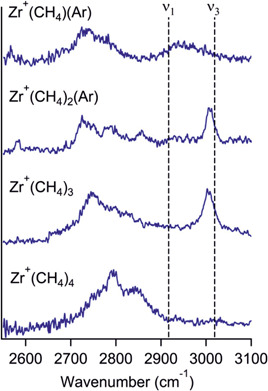
Vibrational spectra of Zr^+^(CH_4_)_*n*_(Ar), (*n* = 1−2) and Zr^+^(CH_4_)_*n*_, (*n* = 3−4) in the C−H stretching region. The symmetric (*ν*_1_) and antisymmetric (*ν*_3_) stretches in bare CH_4_ are shown by the dotted vertical lines. The *y*‐axis shows the normalized photofragment yield. Reprinted with permission from reference Kozubal et al. (2020). Copyright (2020) American Chemical Society [Color figure can be viewed at wileyonlinelibrary.com]

The last methane complex with a metal ion from the yellow part of the periodic table (Figure 1) discussed is that with zirconium. Zirconium seems to be a borderline example as it forms adducts with methane (Figure 7), but the authors also detected dehydrogenation resulting from C–H activation reactions (Kozubal et al., 2020). Inspection of the zirconium complexes with one to four methane molecules reveals that the spectrum of Zr^+^(CH_4_)_4_ is rather similar to that of Ag^+^(CH_4_)_4,_ suggesting similar geometries for these complexes. Hence, the four methane molecules are η^2^ coordinated and fill the first solvation shell symmetrically. For three and two methane molecules, the spectra more resemble the results obtained for the iron complexes, where the methane molecules are η^3^ coordinated. This analogy is well supported by theoretical results obtained for Zr^+^(CH_4_)_2_ and Zr^+^(CH_4_)_3_ which indeed suggest that η^3^ coordination is preferred.

The IR spectra of Zr^+^(CH_4_)(Ar) and Zr^+^(CH_4_)_2_(Ar) contain a weak band below 2600 cm^−1^. The authors computationally investigated various possible isomers. The conclusion was that this spectroscopic feature evidences the presence of the carbene–metal motif. Hence, apart from the dominant zirconium complexes with intact methane molecules, zirconium also forms complexes formulated as [Zr^+^(CH_2_)(H_2_)(Ar)] and [Zr^+^(CH_2_)(H_2_)^+^(CH_4_)(Ar)] (Armentrout & Sievers, 2003).

Recently, the first evidence for methane activation by a metal anion, Pt^−^, has been reported (Liu et al., 2019). Using photoelectron spectroscopy of the mass‐selected [PtCH_4_]^−^ complex, the authors were able to show that this complex corresponded to an activated methyl platinum hydride [H–Pt–CH_3_]^−^ species. The complex had a limited tendency to the elimination of H_2_ molecule(s) which was further suppressed when the experiment was performed with CD_4_ because of the kinetic isotope effect.

### 3.2 [Metal(CH_2_)]^+/−^ complexes

**Figure 8:**
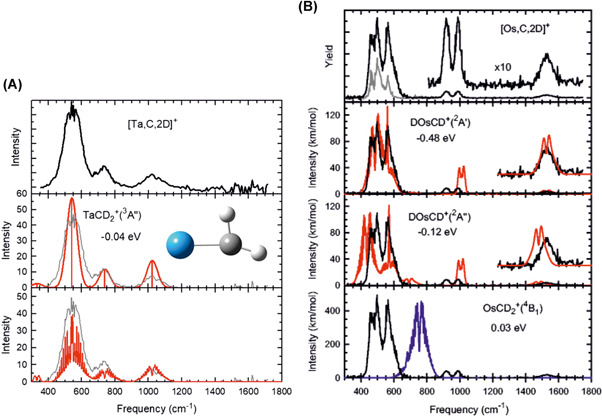
(A) Infrared multiple photon dissociation (IRMPD) spectrum for [Ta,C,2D]^+^ (top, black; middle and bottom, gray) and DFT calculated spectrum for the distorted triplet carbene structure (middle, red) and including rotational contours (bottom, red). The energy relative to the Ta^+^ + CD_4_ reactants is shown along with the structure of the ^3^A″ state. Adapted with permission from reference Owen et al. (2018). Copyright (2018) AIP Publishing. (B) IRMPD spectra for [Os,C,2D]^+^ (top panel, black: high IR laser intensity; gray: low IR laser intensity) and the DFT calculated spectra including rotational band structure for the doublet carbyne deuteride (middle traces, red) and quartet carbene (bottom trace, blue). The energies are given relative to the Os^+^ + CD_4_ reactants. The lowest frequency band of the DOsCD^+^ (^2^A′) species has been blue‐shifted by 30 cm^−1^. Adapted with permission from reference Armentrout et al. (2018). Copyright (2018) American Chemical Society [Color figure can be viewed at wileyonlinelibrary.com]

Middle and late 5d transition metal cations (the green elements in Figure 1) can bring about the C–H activation and thus continue on the reaction pathway towards dehydrogenation (Scheme 1) (Armentrout, 2017; Dietl et al., 2012; Roithova & Schröder, 2010; Schwarz, 2011, 2014; Schwarz et al., 2017). The detected ion products can correspond either to metal–carbene (M^+^CH_2_) ions or to hydrido metal–carbyne (HM^+^CH) ions. Ion spectroscopy can help in determining the structures as demonstrated in a series of papers from the Armentrout group in cooperation with the FELIX team (Table 2). The authors showed that Ta^+^, W^+^, and Pt^+^ react with methane to produce metal carbene cations (Armentrout et al., 2018; Lapoutre et al., 2013; Owen et al., 2018). The individual metal cations affect the structure of the carbene complexes by their electronic structure. Ta^+^ and W^+^ seek additional stabilization of the complex by agostic interaction with one of the C–H bonds. The structure of the metal carbene complex for these elements is thus distorted as shown in Figure 8A. In contrast, the platinum carbene complex has C_2V_ symmetry. The platinum cation cannot be stabilized by the agostic interaction, because it has all d orbitals occupied.

The metal carbene complexes can be also prepared indirectly by a reaction with ethylene oxide. Such a pathway allows studying complexes of metal cations that do not dehydrogenate methane at room temperature. An example is a study of [Au,C,2H]^+^ prepared by reacting Au^+^ with ethylene oxide. It was found that [Au,C,2H]^+^, like Pt^+^ and the early 5d ions, adopts a carbene structure. However, theoretically it was established that the metal–methylidene bond changed from a double bond common to the other 5d metal ions to a dative bond for Au^+^ (Armentrout et al., 2019).

**Table 2:** Ion spectroscopy of metal carbene and metal carbyne hydride cationic complexes^a^

		Spectral range (cm^−1^)	References
Ta^+^	[Ta(CH_2_)]^+^	300–3300	Lapoutre et al. (2013)
	[Ta(CD_2_)]^+^	300–1800	Owen et al. (2018)
	[Ta(CH_2_)Ar]^+^	600‐1800	Bakker et al. (2021)
W^+^	[W(CH_2_)]^+^	300–3300	Lapoutre et al. (2013)
	[W(CD_2_)]^+^	300–1800	Owen et al. (2018)
Os^+^	[HOs(CH)]^+^	300–1800	Armentrout et al. (2018)
Ir^+^	[(H)Ir(CH)]^+^, minor [Ir(CH_2_)]^+^	300–3300	Lapoutre et al. (2013)
	[(D)Ir(CD)]^+^	300–1800	Owen et al. (2018)
	[(H)Ir(CH)Ar]^+^	600‐1600	Bakker et al. (2021)
Ir^+^	[Ir,C_*n*_,H_*m*_]^+^ (*n* = 3, 4; *m* = 8, 10, 12)	500–1800	Wheeler et al. (2019)
Pt^+^	[(Pt(CH_2_)]^+^	300–3300	Wheeler et al. (2019)
	[(Pt(CD_2_)]^+^	300–1800	Owen et al. (2018)
	[Pt(CH_2_)Ar]^+^	600‐1800	Bakker et al. (2021)
Pt^+^	[Pt(CH_2_)(CH_4_)_*m*_]^+^ (*m* = 1–3)	400–1700	Wheeler et al. (2016)
Au^+^	[Au(CH_2_)]^+^	300–1850	Armentrout et al. (2019)

^a^ All results were obtained by the infrared multiple photon dissociation method.

In contrast to the early and late 5d ions, the Ir^+^ and Os^+^ cations can follow a different reaction pathway, leading from a methyl–metal hydride ion to the hydrido metal carbyne complex under elimination of H_2_. The structures of these complexes have been again clearly identified by their IRMPD spectra (Figure 8b).

The spectra of all [M,C,2H]^+^ structures under investigation exhibited some very broad features, that were first thought to originate from the high IR intensity needed to induce fragmentation in these strongly bound species. Later, it was realized that these relatively small molecular systems have large rotational constants, causing the rotational envelopes of vibrational transitions to fan out over tens of cm^−1^ with remarkable substructure, thereby significantly affecting the appearance of the IRMPD spectra. They also showed that these transitions can be well explained by theoretical calculations (cf. Figure 8) and even further substantiate the spectral assignments. It is also of interest to note that despite the simplicity of the systems studied, the theory is not without its deficiencies in predicting vibrational frequencies, as illustrated by the requirement to shift specific modes to match the spectral structure observed (Figure 8b).

**Figure 9:**
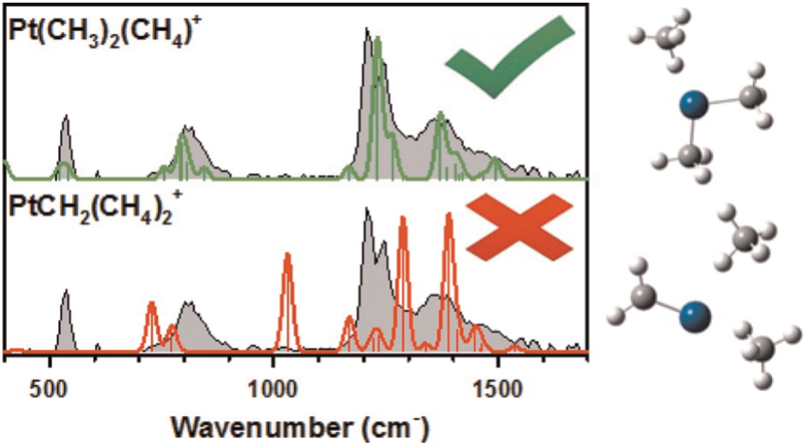
Infrared multiple photon dissociation spectrum for [Pt,3C,10H]^+^ (gray) and DFT (B3LYP/def2‐TZVPPD) calculated spectra for the structures indicated in the figure. Reprinted with permission from reference Wheeler et al. (2016). Copyright (2016) American Chemical Society [Color figure can be viewed at wileyonlinelibrary.com]

For the metal cations that mediate methane dehydrogenation, the next question is how does the reaction continues in the presence of excess methane molecules. This question has been addressed in the case of platinum and iridium cations (Wheeler et al., 2016, 2019). As mentioned above, reaction of the first methane molecule with Pt^+^ forms a C_2V_ symmetrical carbene complex. The interaction with another methane molecule does not lead to the simple formation of an adduct (Figure 9 bottom), but to the activation of the second methane molecule, and transfer of a hydrogen from methane to the carbene group. Hence, the resulting complex bears two methyl groups (Figure 9 top). The authors excluded the possibility of a reaction between the carbon atoms leading to complexes with an ethyl group or another C2‐ligand.

Reacting Ir^+^ with methane predominantly forms a hydrido carbyne structure. No spectra were reported for the adsorption of two methane molecules, but for three methane molecules the spectra indicated the formation of an HIr^+^‐trimethyl structure, and for four methane molecules the spectra evidenced a C–C coupling to form Ir^+^(CH_3_)_2_C_2_H_4_ (Wheeler et al., 2016).

### 3.3 Methane activation by metal clusters

Reactions of methane with metal clusters represent a transition from fundamental studies involving only isolated metal ions towards more realistic catalytic systems based on nanoparticles up to metal surfaces. Metal–metal interactions can lead to the formation of less, but also more reactive sites compared to the isolated metal ions (Albert et al., 1997). The former category of the metal clusters is derived from the metal cations that do not bring about dehydrogenation of methane and that interact with a methane molecule in a η^3^ fashion. Detailed studies of methane reacting with Mg_2_^+^ (Cheng et al., 1997) and various Fe_*n*_^+^ clusters (Ashraf et al., 2015; Copeland et al., 2017) revealed that the clustering did not increase the reactivity in terms of achieving a C–H bond cleavage (Table 3). The methane molecule coordinates to the metal atoms of the clusters in the η^3^‐fashion as reported for the singular metal cations (see above).

**Table 3:** Ion spectroscopy of complexes between metal clusters and methane or products of the cluster–methane reactions

	*n*	*m*	Method	Spectral range (cm^−1^)^a^	References
[Mg_2_(CH_4_)]^+^	2	1	visPD	540–700 nm	Cheng et al. (1997)
[Fe_n_(CH_4_)_*m*_]^+^	2	1–3	IRMPD	2650–3100	Ashraf et al. (2015)
[Fe_n_(CH_4_)_*m*_]^+^	3, 4	1–4		2650–3100	Copeland et al. (2017)
[Ta_4_CH_2_]^+^	4	1	IRMPD	300–1800	Lengyel et al. (2020)
[Pt_ *n* _(CH_4_)]^+^	2–4	1	Ar tagging, IRMPD	650–1600	Harding et al. (2012)
[Au_*n*_(CH_4_)]^+^	2–4	1	IRMPD	200–1800	Lang et al. (2017)

Abbreviations: IRMPD, infrared multiple photon dissociation; visPD, visual photodissociation.

^a^ If not stated otherwise.

A systematic study of the reaction with iron clusters showed that the IR spectra of the [Fe_*n*_(CH_4_)_*m*_]^+^ are dominated by a single band in the range of C–H stretch vibrations. The band is localized at about ∼2800 cm^−1^ which is red shifted with respect to the C–H stretches of the free methane molecule. The small shifts within the series further show two trends. First, the more methane molecules are coordinated to a given cluster, the higher the frequency of the observed band ([Fe_2_(CH_4_)]^+^ (2803 cm^−1^)–[Fe_2_(CH_4_)_2_]^+^ (2829 cm^−1^)–[Fe_2_(CH_4_)_3_]^+^ (2830 cm^−1^)). Secondly, in the series of the [Fe_*n*_(CH_4_)_*n*_]^+^ complexes with one methane molecule per iron atom, the band frequency decreases with increasing cluster size ([Fe_2_(CH_4_)_2_]^+^ (2829 cm^−1^)–[Fe_3_(CH_4_)_3_]^+^ (2809 cm^−1^)–[Fe_4_(CH_4_)_4_]^+^ (2795 cm^−1^)). While the first finding appears intuitive, suggesting that the interaction of the Fe_2_^+^ is charge dominated, which is diluted when more methane molecules bind, the second finding is more important. It shows that the iron effect on the C–H bonds is not primarily due to the electrostatic interaction, but rather due to covalent binding between an iron atom and methane.

Adsorption of methane on Au_n_^+^ (*n* = 2–4) clusters was studied in the 300–1800 cm^−1^ spectral range, so no comparison can be made to the work above (Lang et al., 2017). Nevertheless, the dominant bands indicated absorption of intact methane molecules with the two deformation vibrations *ν*_2_ and *ν*_4_ doubly and triply degenerated and split into several intense bands. Interestingly, weaker bands pointed to the possible coexistence of isomers where a C–H band was activated resulting in the formation a methyl group. Complementary DFT calculations predicted this product to be slightly endothermic with respect to the reactants, albeit within the reach at room temperature.

**Figure 10:**
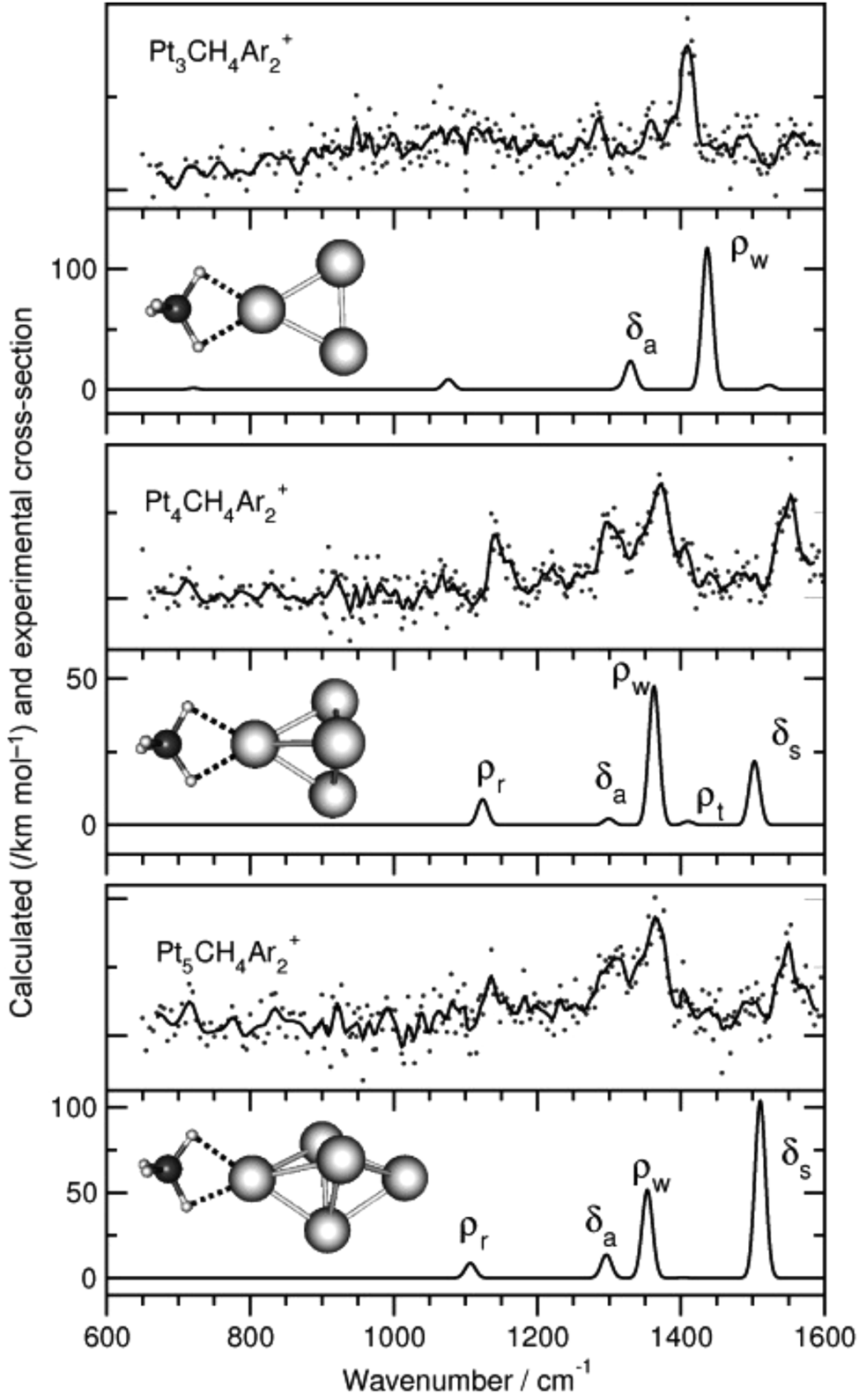
Experimental infrared multiple photon dissociation spectra of Pt_*n*_CH_4_Ar_2_^+^ (*n* = 3–5), monitored by depletion of the parent‐ion signal in all cases (gray points) accompanied by calculated structures, with vibrational mode descriptions, that provide the best match to the experiment are shown for the Pt_*n*_CH_4_^+^ complexes. Reprinted with permission from reference Harding et al. (2012). Copyright (2012) Wiley‐VCH

The next family of metals are those that dehydrogenate methane and yield metal carbene complexes. An example of clusters derived from this family are platinum clusters. As shown above, the platinum cation dehydrogenates methane molecules to form platinum carbene complexes. Platinum anion activates methane as well, however, the reaction ends up in formation of [H–Pt–CH_3_]^−^. The interaction of methane with Pt_*n*_^+^ clusters with *n* = 3–5 leads to rapid dehydrogenation of the methane molecules, but these products were not spectroscopically characterized (Harding et al., 2012). Instead, Fielicke and coworkers managed to form an entrance product by producing [Pt_*n*_Ar]^+^ clusters and forming Pt_*n*_^+ ^methane complexes via the exchange of the Ar atom. In this complex, a methane molecule is coordinated in η^2^ fashion to the cluster and the reaction does not further proceed towards the formation of a carbene complex (Figure 10). The formation of the initial complex by a ligand exchange (here, exchange of Ar by CH_4_) leads to a complex with a smaller internal energy compared to the energy obtained by the direct reaction between the metal (cluster) and CH_4_. The internal energy of the [Pt_*n*_CH_4_]^+^ generated by the ligand exchange was insufficient to promote a C–H bond‐breaking process.

**Figure 11:**
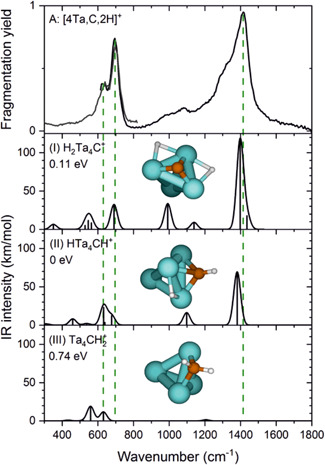
Experimental infrared (IR) multiple photon dissociation spectra (top row) for [4Ta,C,2H]^+^. Calculated (scaled) harmonic spectra at the PBE + D2/TZVP level of theory for (I) a carbide dihydride, (II) a carbyne hydride, and (III) a carbene (Rows 2, 3, and 4, respectively). The relative energies (in eV) of the corresponding structural isomers with respect to isomer II, the carbyne hydride. Adapted with permission from reference Lengyel et al. (2020). Copyright (2020) Wiley‐VCH [Color figure can be viewed at wileyonlinelibrary.com]

The last example is a cluster of tantalum, Ta_4_^+^ (Lengyel et al., 2020). The tantalum cation dehydrogenates methane and forms the carbene complex stabilized by an agostic interaction (see Figure 8A). The Ta_4_^+^ cluster also dehydrogenates methane, but the activation pathway goes even further (Figure 11). The IRMPD spectra and DFT calculations suggest that the carbon atom does not interact with only one tantalum atom, but is rather bound between two of the metal atoms. The carbene structure of the detected [Ta_4_CH_2_]^+^ complex is the least stable isomer and can be excluded based on the experimental IRMPD spectrum. Instead, the spectra are consistent with either a carbide dihydride or carbyne hydride structure of the complex (see Figure 11). The further analysis and experiments with several methane isotopologues confirmed that the experimentally detected complexes can be assigned as a carbide dihydride. This is a clear example, in which a cooperation between metal atoms in a cluster makes a more reactive system than an isolated metal ion alone.

## 4 CONCLUSIONS

This review gives an overview of ion spectroscopic studies that investigate the interaction between metal ions or clusters and methane molecules and the resulting reaction products. The majority of the studies infer the interaction of metals with methane molecules based on the spectral shifts of the observed CH stretching vibrations of CH_4_. The spectral bands and their shifts are usually assigned based on density functional theory calculations. The results reveal the coordination modes of the methane molecules to a metal and clearly show formation of the first and secondary solvation shells. The overview shows different ways of the interaction of the main group and the transition metals with methane molecules. In particular, it shows that the late transition metals tend to coordinate methane in a η^2^ fashion, whereas the metals with low electronegativity interact with methane molecules in a η^3^ fashion.

When metal cations dehydrogenate methane either carbene or carbyne hydride complexes are formed. For these species, comparison to the free methane molecule spectrum loses its meaning, therefore the spectra are necessarily compared to the outcomes of theoretical predictions. In that, researchers were even able to use the rovibrational broadening of vibrational transitions to identify the structures of these small complexes.

Currently, research on the metal–methane interaction gets more oriented towards bridging the gap between the small atomic models and realistic catalytic conditions. Therefore, studies of metal clusters and their interactions with methane molecules start to appear. The studies show that the metals that bind methane mostly via electrostatic interactions (represented by η^3^ coordination), interact with methane in the same way even if present in clusters. For more reactive metals, the formation of clusters can make them even more reactive as shown by the example of a gold or a tantalum cluster. As a future outlook, we expect that ion spectroscopy will shed more light on metal–metal cooperation in methane activation and unravel the ideal compositions of prospective catalysts.
